# Development and validation of prognostic nomograms for adult with papillary renal cell carcinoma: A retrospective study

**DOI:** 10.1016/j.clinsp.2024.100374

**Published:** 2024-05-07

**Authors:** Qingxiang Guo, Sai Li, Jiawei Zhu, Zewei Wang, Zhen Li, Junqi Wang, Rumin Wen, Hailong Li

**Affiliations:** a Department of Urology, the Affiliated Hospital of Xuzhou Medical University, Xuzhou, China; b Department of Neurosurgery, The Second Affiliated Hospital of Anhui Medical University, Hefei, China

**Keywords:** Nomogram, Seer, Papillary renal cell carcinoma, Survival Analysis

## Abstract

**Objective:**

The aim of the study was to create two consensus nomograms for predicting Overall Survival (OS) and Cancer-Specific Survival (CSS) in adults with papillary Renal Cell Carcinoma (pRCC).

**Methods:**

Using the Surveillance, Epidemiology, and End Results databases, a retrospective analysis of 1,074 adults with pRCC from 2004 to 2015 was performed. These patients were then randomly divided into two independent cohorts with a ratio of 7:3 (training cohort: 752; validation cohort: 322). In a retrospective analysis of 752 patients from the training cohort, independent prognostic variables affecting OS and CSS were found. R software was used to create prognostic nomograms based on the findings of Cox regression analysis. The performance of the nomograms was assessed using the Concordance Index (C-index), the Area Under Curve (AUC), a calibration curve, and Decision Curve Analysis (DCA). Data from the 107 postoperative pRCC patients at the Affiliated Hospital of Xuzhou Medical University were used for external validation of the nomogram.

**Results:**

For OS and CSS, the C-indices and AUCs of the training cohort and the validation cohort indicated that the model had excellent discrimination. The DCA demonstrated that the model was clinically applicable, and the calibration curves in the internal and external validations showed that the model's accuracy was high.

**Conclusion:**

The authors developed and validated a prognostic nomogram that accurately predicted the 3-, 5-, and 8-year OS and CSS of adults with pRCC. Clinicians can use this knowledge to direct the clinical management and counseling of patients with pRCC.

## Highlights

• Radiotherapy benefits prognosis in papillary renal cell carcinoma.
• A precise nomogram predicts survival in pRCC patients.
• External validation confirms the nomogram's applicability to Chinese pRCC.

## Introduction

Renal Cell Carcinoma (RCC) is a type of malignant tumor that arises in the epithelium of the renal tubules.^1^ In urological malignancies, RCC is the third-most prevalent malignant tumor, behind prostate cancer and bladder cancer, and comprises 3 % of all female and 5 % of all male cancers.^2^^,^^3^ RCC patients have a poor prognosis, and about 30 % of them already have metastases when they are diagnosed, preventing them from being surgically treated.^4^ Regarding all kidney neoplasms, the second-most frequent type of renal cell cancer is papillary Renal Cell Carcinoma (pRCC).^5^ Although a better prognosis exists for pRCC than for clear cell renal cancer, holistic consideration of clinical data such as tumor stage and subtype is required for the best follow-up or treatment.^6^ Due to the low incidence and few instances in an individual center, pRCC has received minimal attention. Furthermore, there is no specific prediction model to assess the prognoses of pRCC patients. Therefore, to direct clinical treatment, investigation into the variables affecting pRCC patient prognoses is crucial.

The Tumor-Node-Metastasis (TNM) staging system developed by the American Joint Committee on Cancer (AJCC) is the most commonly used to assess the prognoses of RCC patients.^7^ However, its main drawbacks are a lack of accuracy, the exclusion of other criteria (such as age), and an inadequate ability to predict patient survival,^8^ so patients with pRCC need a customized prediction model.

Nomograms have been increasingly used in cancer treatments as a prediction technique.^9^ They comply with standards for an integrated model, contribute to the development of customized medicine, and are practical for physicians to use in prognosis prediction.^10^^,^^11^ In the present study, therefore, the authors created a nomogram to predict the Overall Survival (OS) and Cancer-Specific Survival (CSS) of adults with Prcc. The authors compared it to TNM staging and Fuhrman grading in terms of its ability to predict patient survival. Furthermore, the authors evaluated the performance of nomograms and performed internal and external validations.

## Materials and methods

### *Data source and patients*

The authors used the Surveillance, Epidemiology, and End Results (SEER)*stat software to identify 1074 suitable adults who were diagnosed with pRCC between 2004 and 2015 from the SEER database, which contains clinicopathological and distinct prognostic data. The inclusion criteria were as follows: (i) Papillary renal cell carcinoma as the only or first primary tumor that was confirmed by histology, (ii) Patients with surgically removed malignancies, and (iii) Active follow-up to ensure reliable patient status. The exclusion criteria were as follows: (i) Patients who were older than 85 years and younger than 18 years; (ii) Patients with insufficient follow-up data, such as missing information on ethnicity, surgery type, lymph node status, tumor stage, pathological grade of the tumor, and adjuvant therapy; and (iii) Following initial diagnosis, the patient died within 1 month.

**Fig. 1:**
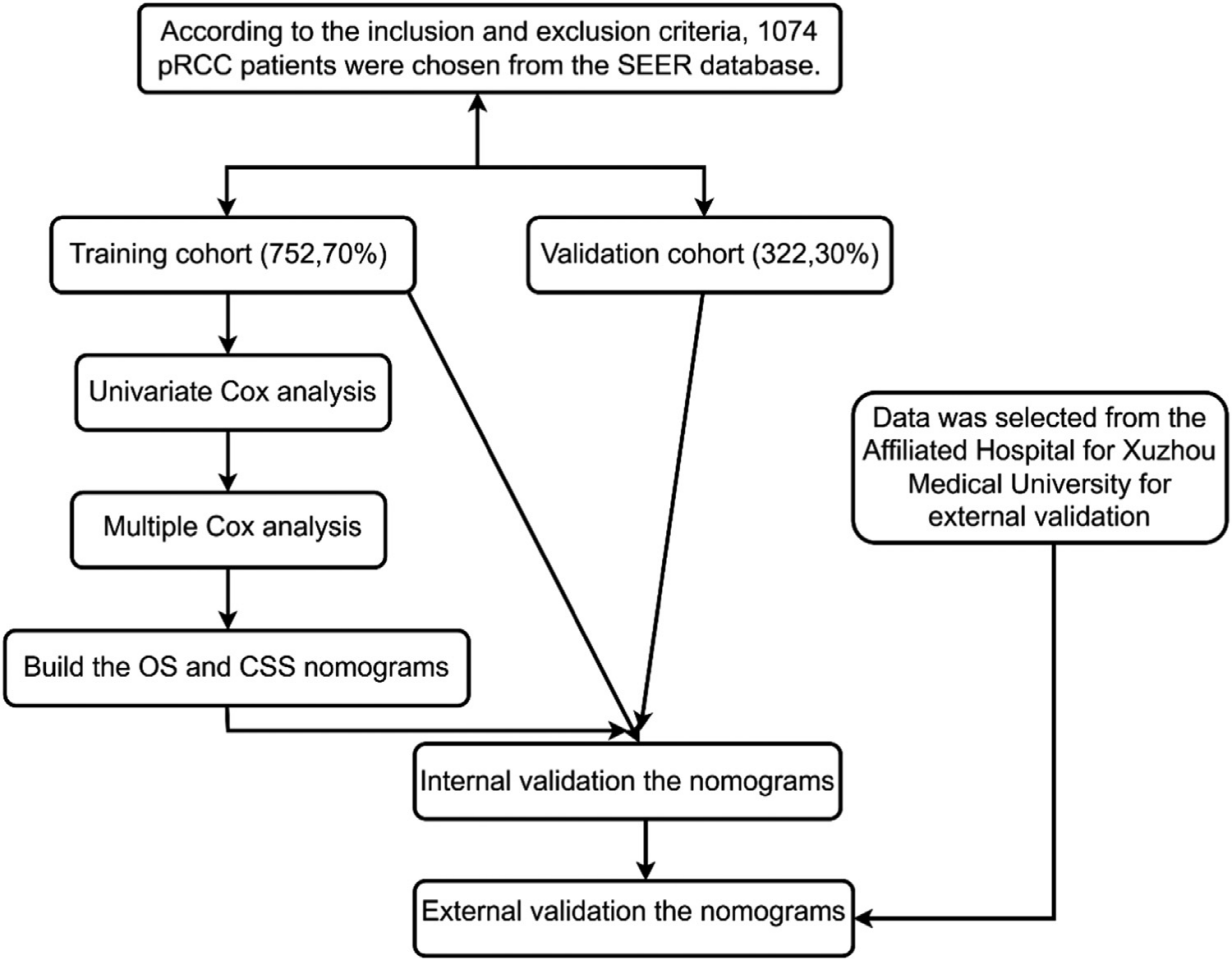
Flowchart of the study (By Figdraw). Abbreviations: PRCC, Papillary Renal Cell Carcinoma; SEER, Surveillance, Epidemiology, and End Results; OS, Overall Survival; CSS, Cancer-Specific Survival.

Ultimately, 1074 patients were enrolled in this study. To develop a nomogram survival prediction model and identify independent predictors of OS and CSS, the authors divided the 1074 patients into two cohorts: a training cohort (to identify independent predictors of OS and CSS and create a nomogram survival prediction model) and a validation cohort (to internally validate our model). Additionally, from 2004 to 2015, the authors collected clinical information on 107 patients who were pathologically diagnosed with pRCC at the Affiliated Hospital of Xuzhou Medical University, in order to externally verify the nomogram's functionality. The studies involving human participants were reviewed and approved by the Ethics Committee of the Affiliated Hospital of Xuzhou Medical University (XYFY2021-KL092–02). Fig. 1 shows the study flowchart. The study has been carried out in accordance with the STROBE Statement.

### *Study variables*

Clinical factors covered in the SEER database included age at diagnosis, race, sex, marital status, laterality, months from diagnosis to treatment, pathological grade of the tumor, tumor size (mm), median annual family income, AJCC stage, operation strategy, TNM stage, chemotherapy, and radiotherapy. Pathological TNM staging was performed using the AJCC TNM staging system, sixth edition; and tumors were graded according to their histological characteristics, which included grade 1 (well differentiated), grade 2 (moderately differentiated), grade 3 (poorly differentiated), and grade 4 (undifferentiated). The OS time represented the period from the time of patient diagnosis to the date of death or the date at which records were destroyed, while the CSS time was the time between diagnosis and death. The death date or the final follow-up in November 2020 served as the end of the study.

### *Statistical analysis*

All patients who met the criteria were randomly assigned to the training (*n* = 752) or validation (*n* = 322) groups in a 7:3 ratio. X-Tile software was used to find the best cut-off points for age at diagnosis, a month from diagnosis to therapy, and tumor size (Version 3.6.1, Yale University, New Haven, CT, USA).^12^ In the training cohort, prognostic factors were screened out using univariate Cox regression, and independent risk factors were examined using multivariate Cox regression. Additionally, the authors simultaneously recorded the hazard ratio and 95 % Confidence Interval (95 % CI).

Adults with pRCC had the 3-, 5-, and 8-year OS and CSS predicted using a nomogram based on independent risk factors identified by univariate and multivariate Cox regression analyses. Each variable was dispersed on the nomogram in accordance with its weight, to produce different lines, and each variable point corresponded to one point. The total points of the nomogram ‒ that is, the sum of the points for each of the variables ‒ were used to calculate survival at different times.

Internal and external validations were used to test the performance of the nomogram. Externally validated patient data were gathered from the Affiliated Hospital of Xuzhou Medical University. The nomogram was then verified using a series of verification methods, such as the C-index, the Receiver Operating Characteristic (ROC) curve, and a calibration curve. The calibration curve, which was used to assess the connections between the observed and real values, was the principal tool used to test the accuracy of the nomogram. In addition, the prognostic abilities of the nomogram, TNM stage, and pathological grade were assessed using ROC curves.

The clinical effectiveness of the model was evaluated using Decision Curve Analysis (DCA) according to the net gain under each risk threshold.^13^ The clinical applicability of the nomogram was further assessed using the DCA. Then, based on each patient's nomogram score, the authors divided the patients into low-risk, intermediate-risk, and high-risk groups. The Kaplan-Meier curve was then used to compare variations in survival among various risk groups.

R software (R Foundation for Statistical Computing, Vienna, Austria) and SPSS (IBM, Armonk, NY, USA) were used for all statistical analyses (Version 4.2.1). Statistics were deemed significant at *p* < 0.05.

## Results

### *Clinical features*

Finally, 1074 adult pRCC patients who met the inclusion and exclusion criteria were chosen at random from the SEER database and divided into the training cohort (*n* = 752) and validation cohort (*n* = 322) in a 7:3 ratio. In the overall cohort, there were 720 married patients, 880 Caucasian patients, and 753 male patients. There were 565 patients (52.6 %) whose tumors were on the left side. These patients had higher tumor T-stages (70.9 % of patients with T3 or T4 stage) and pathology grades (about 76.2 % of patients have a grade of 3 or higher). A total of 144 (13.4 %) tumors were stage I, 78 (7.26 %) tumors were stage II, 313 (29.1 %) tumors were stage III, and 539 (50.24 %) tumors were stage IV. A total of 85 patients (7.91 %) underwent partial nephrectomy, and 989 (92.1 %) patients underwent radical nephrectomy. A total of 147 patients (13.7 %) received radiation before or after surgery, while 326 patients (30.4 %) received chemotherapy. There was no appreciable difference between the two groups according to patient data from the training and validation sets (Table 1).

**Table 1:** Patients’ demographics and clinicopathological characteristics.

Variables	Total (*n* = 1074)	Training set, *n* = 752 (70 %)	Validation set, *n* = 322 (30 %)	p
Age of diagnosis	61 (53.0‒70.00)	61 (53.5‒70.50)	62 (53.0‒70.0)	0.845
Race				0.305
White	880 (81.9 %)	619 (82.3 %)	261 (81.1 %)	
Non-White	194 (18.1 %)	133 (17.7 %)	61 (18.9 %)	
Sex				0.584
Female	321 (29.9 %)	221 (29.4 %)	100 (31.1 %)	
Male	753 (70.1 %)	531 (70.6 %)	222 (68.9 %)	
Marital status				0.961
Married	720 (67.0 %)	504 (67.0 %)	216 (67.1 %)	
Single	161 (15.0 %)	114 (15.2 %)	47 (14.6 %)	
Divorced/Wido-wed	193 (18.0 %)	134 (17.8 %)	59 (18.3 %)	
Laterality				0.472
Left	565 (52.6 %)	401 (53.3 %)	164 (50.9 %)	
Right	509 (47.4 %)	351 (46.7 %)	158 (49.1 %)	
Months from diagnosis to treatment				0.906
< 1	621 (57.8 %)	435 (57.8 %)	186 (57.8 %)	
1‒2	303 (28.2 %)	210 (27.9 %)	93 (28.9 %)	
> 2	150 (14.0 %)	107 (14.2 %)	43 (13.4 %)	
T Stage				0.222
T1	184 (17.1 %)	139 (18.5 %)	45 (14.0 %)	
T2	129 (12.0 %)	93 (12.4 %)	36 (11.2 %)	
T3	573 (53.4 %)	388 (51.6 %)	185 (57.5 %)	
T4	188 (17.5 %)	132 (17.6 %)	56 (17.4 %)	
N Stage				0.062
N0	761 (70.9 %)	550 (73.1 %)	211 (65.5 %)	
N1	171 (15.9 %)	109 (14.5 %)	62 (19.3 %)	
N2	115 (10.7 %)	72 (9.6 %)	43 (13.4 %)	
Nx	27 (2.5 %)	21 (2.8 %)	6 (1.9 %)	
M Stage				0.145
M0	651 (60.6 %)	451 (60.0 %)	200 (62.1 %)	
M1	418 (38.9 %)	296 (39.4 %)	122 (37.9 %)	
Mx	5 (0.5 %)	5 (0.6 %)	0 (0.0 %)	
AJCC Stage				0.198
Ⅰ	144 (13.4 %)	109 (14.5 %)	35 (10.9 %)	
Ⅱ	78 (7.3 %)	54 (7.2 %)	24 (7.5 %)	
Ⅲ	313 (29.1 %)	207 (27.5 %)	106 (32.9 %)	
Ⅳ	539 (50.2 %)	382 (50.8 %)	157 (48.8 %)	
Grade				0.995
Ⅰ	16 (1.5 %)	11 (1.5 %)	5 (1.6 %)	
Ⅱ	45 (4.2 %)	31 (4.1 %)	14 (4.3 %)	
Ⅲ	188 (17.5 %)	134 (17.8 %)	54 (16.8 %)	
Ⅳ	631 (58.8 %)	441 (58.6 %)	190 (59.0 %)	
Unknow	194 (18.1 %)	135 (18.0 %)	59 (18.3 %)	
Tumor Size	90 (61–123)	90 (60–120)	90 (62–126)	0.755
Operation				0.176
Partial	85 (7.9 %)	65 (8.6 %)	20 (6.2 %)	
Radical	989 (92.1 %)	687 (91.4 %)	302 (93.8 %)	
Radiation				0.709
No	927 (86.3 %)	651 (86.6 %)	276 (85.7 %)	
Yes	147 (13.7 %)	101 (13.4 %)	46 (14.3 %)	
Chemotherapy				0.293
No/Unknown	748 (69.6 %)	531 (70.6 %)	217 (67.4 %)	
Yes	326 (30.4 %)	221 (29.4 %)	105 (32.6 %)	
Median annual family income, (median US dollars*)				0.370
< 35,000	16 (1.5 %)	11 (1.5 %)	5 (1.6 %)	
35,000‒75,000	756 (70.4 %)	539 (71.7 %)	217 (67.4 %)	
> 75,000	302 (28.1 %)	202 (26.9 %)	100 (31.1 %)	

T Stage, Tumor Stage; N Stage, Node Stage; M Stage, Metastasis Stage; AJCC, American Joint Committee on Cancer; Ref, Reference.

**Fig. 2:**
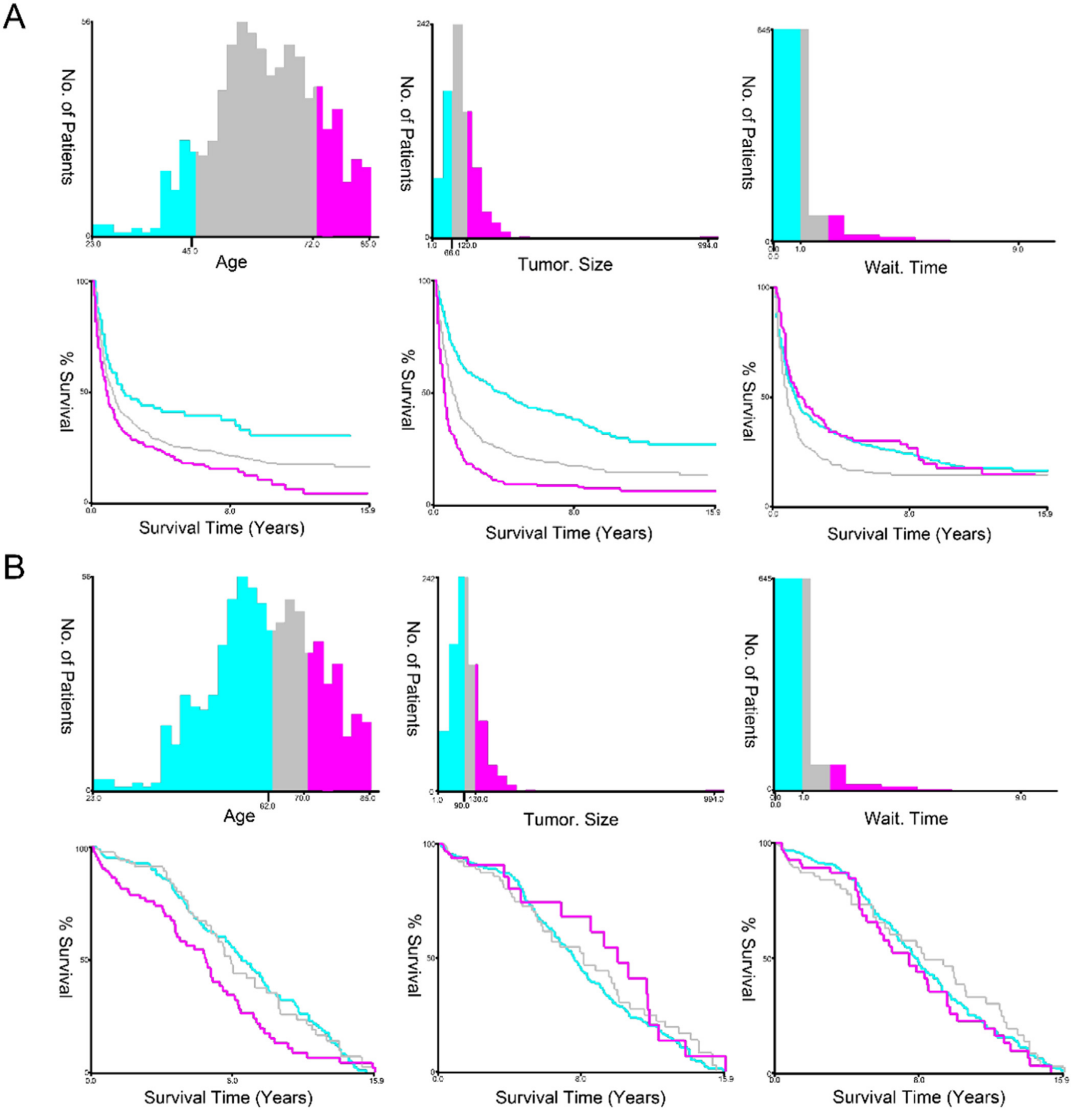
X-tile stratification. (A) The optimum cut-off values of age at diagnosis were 46 and 72 years for OS; The optimal cutoff values of tumor size were 68 mm and 120 mm for OS; the optimal cutoff value in months from diagnosis to treatment was 1 month in OS. (B) The optimum cut-off values of age at diagnosis were 63 and 70 years for CSS. The optimal cut-off values of tumor size were 91 mm and 130 mm for CSS; The optimal cut-off values of month from diagnosis to treatment was 1 month for CSS. All p-values of the corresponding Kaplan-Meier curves were < 0.05.

According to the following cut-off criteria, the authors stratified several variables using the X Tile software. OS: age at diagnosis (≤ 45 years, 46−72 years, ≥ 73 years); tumor size (≤ 66 mm, 67−120 mm, ≥121 mm); month from diagnosis to therapy (< 1 month; 1 − 2 months; ≥ 2 months). CSS: age at diagnosis (≤ 62 years, 63−70 years, ≥ 71 years); tumor size (≤ 90 mm, 91−130 mm, ≥ 131 mm); month from diagnosis to therapy (< 1 month; 1 − 2 months; ≥ 2 months) (Fig. 2).

### *Univariate and multivariate COX regression analyses*

Using univariate and multivariate Cox regression analyses, the prognostic factors for OS and CSS were investigated. The initial 16 factors were first evaluated using univariate COX regression analysis. The results indicated that age at diagnosis, laterality, T stage, N stage, M stage, AJCC stage, grade, tumor size, operation strategy, radiotherapy, and chemotherapy were factors affecting the OS. The following step used multivariate Cox regression analysis to filter the previously mentioned 11 variables. Finally, the authors found that age at diagnosis, T stage, N stage, M stage, AJCC stage, grade, tumor size, and radiotherapy were independent predictors for OS (Table 2).

**Table 2:** Univariate and multivariate analyses of overall survival for training group.

**Variables**	**Univariate analyses hazard ratios (95 % CI)**	**p**	**Multivariate analyses hazard ratios (95 % CI)**	**p**
Age of diagnosis				
≤ 45	Ref.		Ref.	
46–72	1.46 (1.08, 1.96)	0.012	1.45 (1.06, 1.96)	0.018
≥ 73	1.88 (1.35, 2.61)	<0.001	2.30 (1.63, 3.23)	<0.001
Race				
White	Ref.			
Non-White	1.04 (0.74, 1.45)	0.831		
Sex				
Female	Ref.			
Male	1.15 (0.96, 1.37)	0.129		
Marital status				
Married	Ref.			
Single	0.91 (0.72, 1.15)	0.429		
Divorced/Widowed	1.10 (0.89, 1.35)	0.399		
Laterality				
Left	Ref.		Ref.	
Right	0.84 (0.71, 0.98)	0.030	0.93 (0.79, 1.10)	0.400
Median annual family income, (median US dollars*)				
< 35,000	Ref.			
35,000‒75,000	0.49 (0.27, 0.89)	0.484		
> 75,000	0.55 (0.30, 1.02)	0.057		
Months from diagnosis to treatment				
< 1	Ref.			
1	1.34 (1.12, 1.61)	0.625		
> 1	0.92 (0.72, 1.17)	0.479		
T Stage				
T1	Ref.		Ref.	
T2	1.60 (1.14, 2.24)	0.006	1.35 (0.75, 2.44)	0.323
T3	2.92 (2.27, 3.77)	<0.001	1.90 (1.19, 3.04)	0.007
T4	5.38 (4.01, 7.21)	<0.001	2.80 (1.69, 4.65)	<0.001
N Stage				
N0	Ref.		Ref.	
N1	2.11 (1.68, 2.63)	<0.001	1.67 (1.31, 2.13)	<0.001
N2	2.60 (2.00, 3.37)	<0.001	1.57 (1.18, 2.09)	0.002
Nx	4.70 (3.00, 7.37)	<0.001	2.90 (1.75, 4.78)	<0.001
M Stage				
M0	Ref.		Ref.	
M1	2.49 (2.10, 2.94)	<0.001	1.48 (1.09, 2.01)	0.012
Mx	4.26 (1.75, 10.34)	0.001	0.78 (0.29, 2.12)	0.627
AJCC Stage				
Ⅰ	Ref.		Ref.	
Ⅱ	1.63 (1.05, 2.54)	0.031	0.95 (0.46, 1.95)	0.012
Ⅲ	2.77 (2.00, 3.83)	<0.001	1.12 (0.64, 1.95)	0.028
Ⅳ	5.79 (4.26, 7.87)	<0.001	1.45 (0.80, 2.63)	0.221
Grade				
Ⅰ	Ref.		Ref.	
Ⅱ	1.91 (0.55, 6.66)	0.308	1.99 (0.57, 7.00)	0.282
Ⅲ	4.70 (1.49, 14.83)	0.008	2.92 (0.92, 9.32)	0.070
Ⅳ	6.26 (2.01, 19.53)	0.002	3.25 (1.03, 10.26)	0.044
Unknown	5.14 (1.63, 16.21)	0.005	3.53 (1.11, 11.22)	0.033
Tumor Size (mm)				
≤ 66	Ref.		Ref.	
68‒120	1.80 (1.47, 2.20)	<0.001	1.16 (0.90, 1.49)	0.257
≥ 122	2.89 (2.30, 3.61)	<0.001	1.74 (1.31, 2.29)	<0.001
Surgery				0.119
Partial	Ref.		Ref.	
Total	1.95 (1.40, 2.72)	<0.001	0.77 (0.53, 1.12)	0.166
Radiation				
No	Ref.		Ref.	
Yes	1.95 (1.55, 2.46)	<0.001	1.44 (1.13, 1.85)	0.004
Chemotherapy				
No/Unknown	Ref.		Ref.	
Yes	1.75 (1.46, 2.10)	<0.001	0.94 (0.77, 1.14)	0.514

95 % CI, 95 % Confidence Interval.

Similarly, the authors performed univariate and multivariate Cox analyses of CSS and identified independent predictors, including T stage, N stage, M stage, AJCC stage, tumor size, and radiotherapy (Table 3).

**Table 3:** Univariate and multivariate analyses of cancer-specific survival for training group.

**Variables**	**Univariate analyses hazard ratios (95 % CI)**	**p**	**Multivariate analyses hazard ratios (95 % CI)**	**p**
Age of diagnosis				
≤ 62	Ref.			
63‒70	1.35 (0.99, 1.84)	0.055		
≥ 71	1.52 (1.08, 2.15)	0.017		
Race				
White	Ref.			
Non-White	0.84 (0.63, 1.13)	0.491		
Sex				
Female	Ref.			
Male	1.20 (0.99, 1.45)	0.070		
Marital status				
Married	Ref.			
Single	0.84 (0.65, 1.09)	0.184		
Divorced/Widowed	1.04 (0.83, 1.31)	0.709		
Laterality				
Left	Ref.			
Right	0.85 (0.71, 1.01)	0.059		
Median annual family income, (median US dollars*)				
< 35,000	Ref.			
35,000‒75,000	0.54 (0.28, 1.04)	0.065		
> 75,000	0.64 (0.33, 1.25)	0.192		
Months from diagnosis to treatment				
< 1	Ref.			
1	1.39 (1.14, 1.68)	0.107		
> 1	0.86 (0.66, 1.12)	0.265		
T Stage				
T1	Ref.		Ref.	
T2	1.99 (1.37, 2.89)	<0.001	1.25 (0.69, 2.27)	0.464
T3	3.53 (2.63, 4.74)	<0.001	1.77 (1.10, 2.86)	0.019
T4	6.40 (4.59, 8.93)	<0.001	2.43 (1.45, 4.09)	<0.001
N Stage				
N0	Ref.		Ref.	
N1	2.35 (1.86, 2.96)	<0.001	1.80 (1.40, 2.32)	<0.001
N2	2.78 (2.11, 3.65)	<0.001	1.57 (1.17, 2.11)	0.003
Nx	5.07 (3.20, 8.05)	<0.001	2.87 (1.71, 4.82)	<0.001
M Stage				
M0	Ref.		Ref.	
M1	2.83 (2.37, 3.38)	<0.001	1.54 (1.11, 2.12)	0.009
Mx	5.02 (2.06, 12.21)	<0.001	0.91 (0.33, 2.50)	0.860
AJCC Stage				
Ⅰ	Ref.		Ref.	
Ⅱ	2.27 (1.35, 3.81)	0.002	1.18 (0.54, 2.56)	0.685
Ⅲ	3.74 (2.50, 5.59)	<0.001	1.39 (0.75, 2.59)	0.045
Ⅳ	8.46 (5.76, 12.42)	<0.001	1.86 (0.96, 3.58)	0.001
Grade				
Ⅰ	Ref.		Ref.	
Ⅱ	2.36 (0.53, 10.56)	0.260	2.71 (0.60, 12.21)	0.196
Ⅲ	5.72 (1.41, 23.23)	0.015	3.43 (0.84, 14.06)	0.087
Ⅳ	7.96 (1.98, 32.00)	0.004	3.76 (0.93, 15.23)	0.064
Unknown	5.79 (1.43, 23.54)	0.014	3.86 (0.94, 15.80)	0.061
Tumor Size				
≤ 90	Ref.		Ref.	
91‒130	2.46 (1.90, 3.17)	<0.001	1.49 (1.10, 2.03)	0.010
≥ 131	4.13 (3.12, 5.47)	<0.001	2.30 (1.63, 3.23)	<0.001
Surgery				0.432
Partial	Ref.		Ref.	
Total	2.33 (1.58, 3.44)	<0.001	0.75 (0.49, 1.16)	0.196
Radiation				
No	Ref.		Ref.	
Yes	1.95 (1.55, 2.46)	<0.001	1.49 (1.15, 1.92)	0.002
Chemotherapy				
No/Unknown	Ref.		Ref.	
Yes	1.75 (1.46, 2.10)	<0.001	0.96 (0.78, 1.19)	0.731

### *Construction of 3-, 5-, and 8-year nomograms of OS and CSS*

**Fig. 3:**
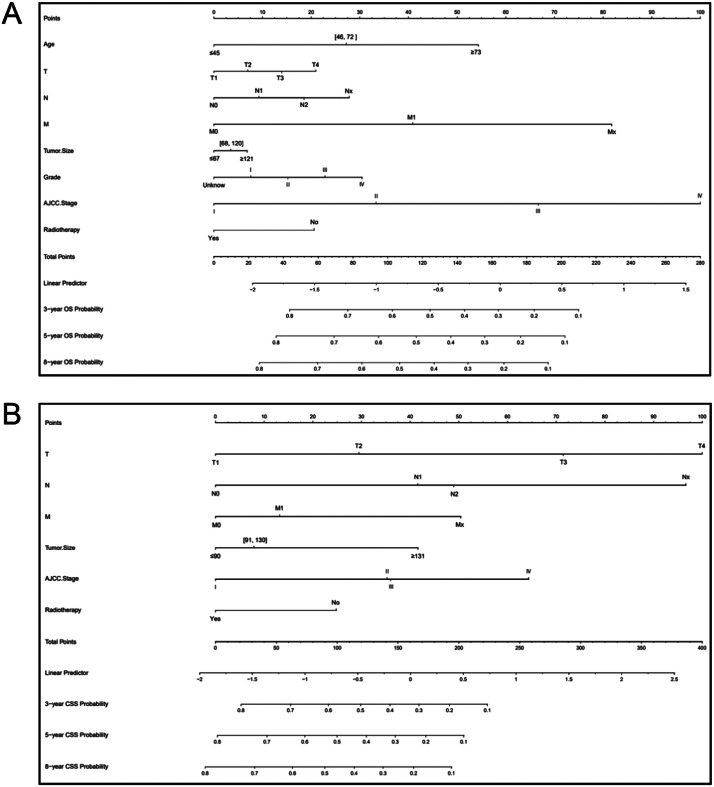
Nomogram for 3-, 5-, and 8-year OS (A) and CSS (B) of adult patients with papillary renal carcinoma.

The authors constructed nomograms of OS and CSS for adults with pRCC according to the screened variables (Fig. 3). According to the nomogram, the AJCC stage was the most important predictive indicator for OS, followed by the M stage and age at diagnosis. The most important factor in the prognosis for CSS was the T stage, which was followed by the N stage and the AJCC stage. The authors also found that the patient's risk of death increased with age; the higher the AJCC stage (T stage, N stage, and M stage), the higher the risk of death. Patients without radiotherapy had a lower survival than those receiving radiotherapy.

### *Performance and validation of the nomogram*

According to the aforementioned established nomogram, the authors initially determined the model's precision using the C-index. A high C-index denoted a high level of discrimination in the model. For OS, the C-indices of the training and validation sets were 0.735 and 0.696, respectively, which were higher than the TNM stage (0.662, 0.6210 and tumor grade (0.567, 0.539). For CSS, the C-indices for training and validation sets were 0.738 and 0.695, respectively, which were likewise higher than the TNM stage (0.666, 0.623) and tumor grade (0.571, 0.542).

**Fig. 4:**
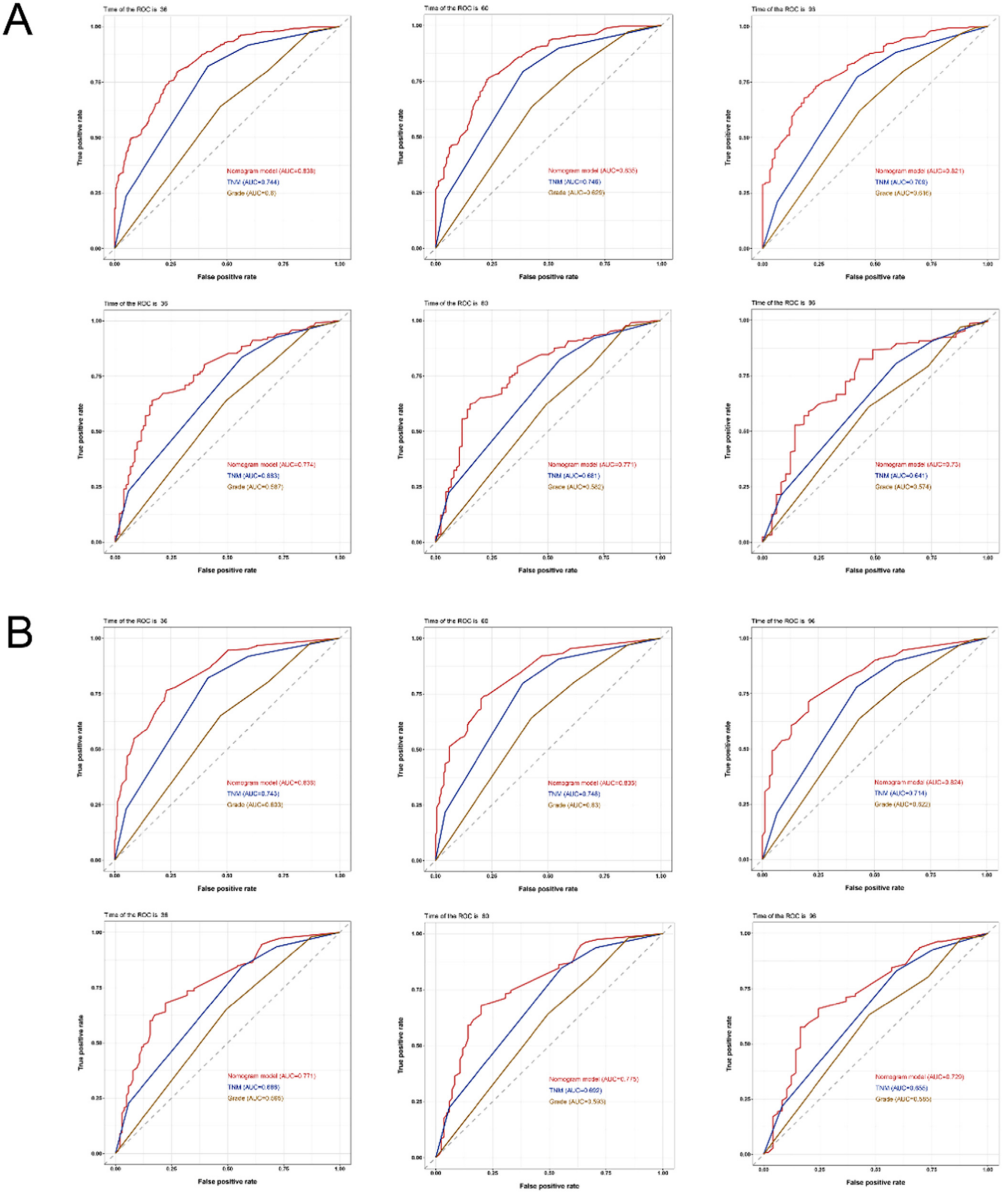
The receiver operating characteristic curves of the nomogram, TNM stage, and tumor grade. (A) The AUCs of the nomogram, TNM stage, and tumor grade for the prediction of prognoses at 3, 5, and 8 years in the training cohort (upper) and validation cohort (lower) for overall survival. (B) The AUCs of the nomogram, TNM stage, and tumor grade to predict the prognoses at 3, 5, and 8 years in the training cohort (upper) and validation cohort (lower) for cancer-specific survival. TNM, Tumor-Node-Metastasis; AUCs, Area Under Curves.

The AUC was therefore used to confirm the model's discriminating abilities. In comparison to the C-index, the trend of model discrimination over time was more clearly displayed by the time-dependent AUC. For the training set, the AUCs for predicting OS were 0.838, 0.835, and 0.821 at 3, 5, and 8 years, respectively, which were significantly higher than the TNM stages (0.744, 0.746, 0.709) and tumor grade (0.600, 0.629, 0.616). For the validation set, the AUCs for predicting OS were 0.774, 0.771, and 0.730 at 3, 5, and 8 years, respectively, which were also higher than the TNM stage (0.683, 0.681, 0.641) and tumor grade (0.587, 0.582, 0.574). For CSS, whether using the training or validation set, the results of the 3-, 5-, and 8-year nomogram AUCs were still higher than those of the TNM stage and tumor grade (Fig. 4), indicating the high levels of discrimination of the model.

**Fig. 5:**
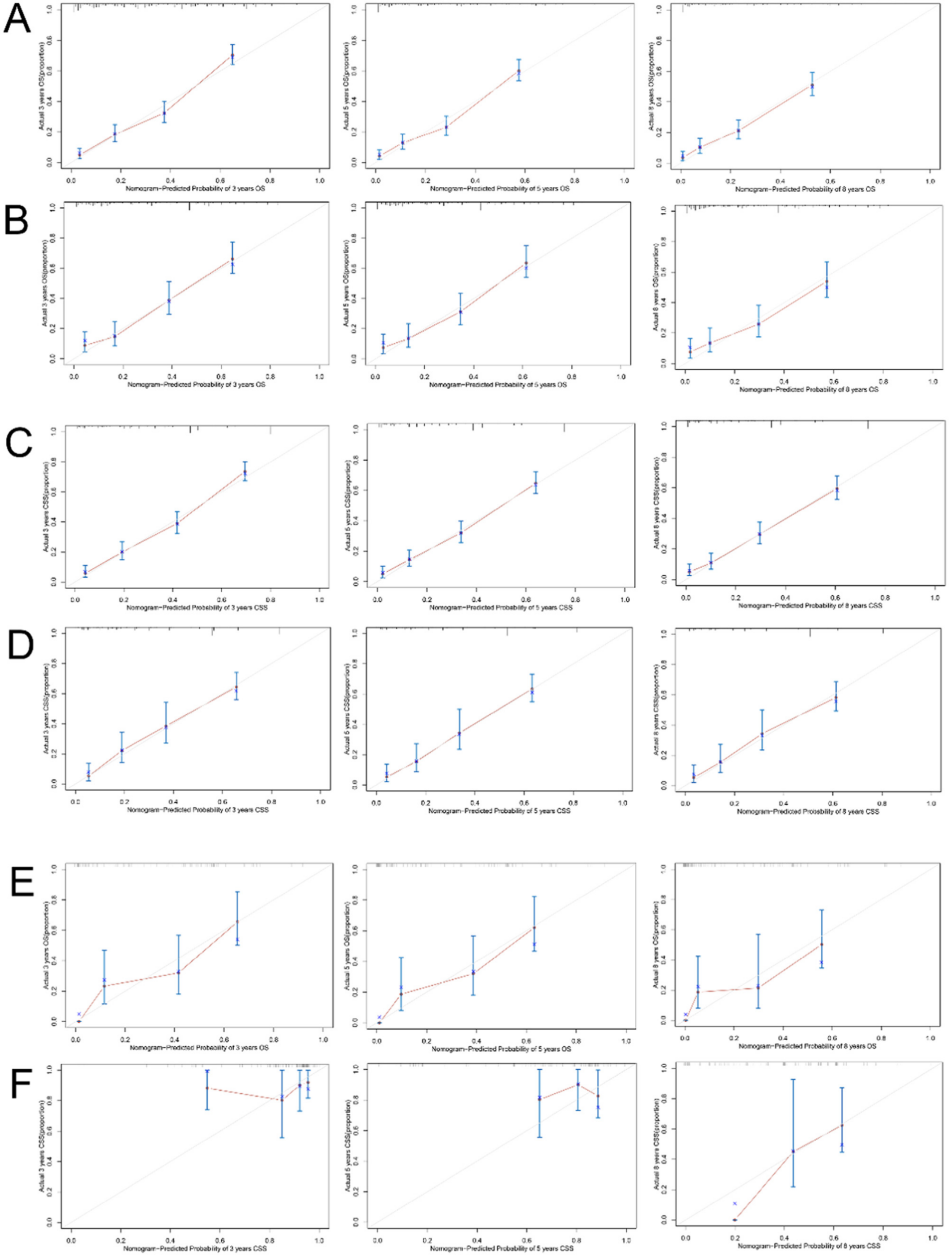
Calibration curves of the nomogram for OS. The 3-, 5-, and 8-year calibration curves for the OS nomogram in the training cohort (A) and validation cohort (B). Calibration curves of the nomogram for CSS. The 3-, 5-, and 8-year calibration curves for the CSS nomogram in the training cohort (C) and validation cohort (D). Calibration curves for the nomogram in the external validation cohort. The 3-, 5-, and 8-year calibration curves for the OS nomogram (E) and CSS nomogram (F) in the external validation cohort.

The accuracy of the model was then verified using calibration curves. The 3-, 5-, and 8-year OS calibration curves were close to the gray line on the diagonal of the training and validation cohorts’ actual survival outcomes (Fig. 5A, B). Furthermore, in the training and validation cohorts, the predicted survivals from the nomogram and the actual survival for CSS agreed to a large extent (Fig. 5C, D). These results showed that the predicted training and verification cohort's actual survival probabilities and results were accurate.

For external validation, the authors also collected clinical information on 107 adults who were pathologically diagnosed with pRCC at the Affiliated Hospital of Xuzhou Medical University. The calibration curves showed good agreement between the 3-, 5-, and 8-year OS and CSS and the diagonal gray line of the actual survivals (Fig. 5E, F).

**Fig. 6:**
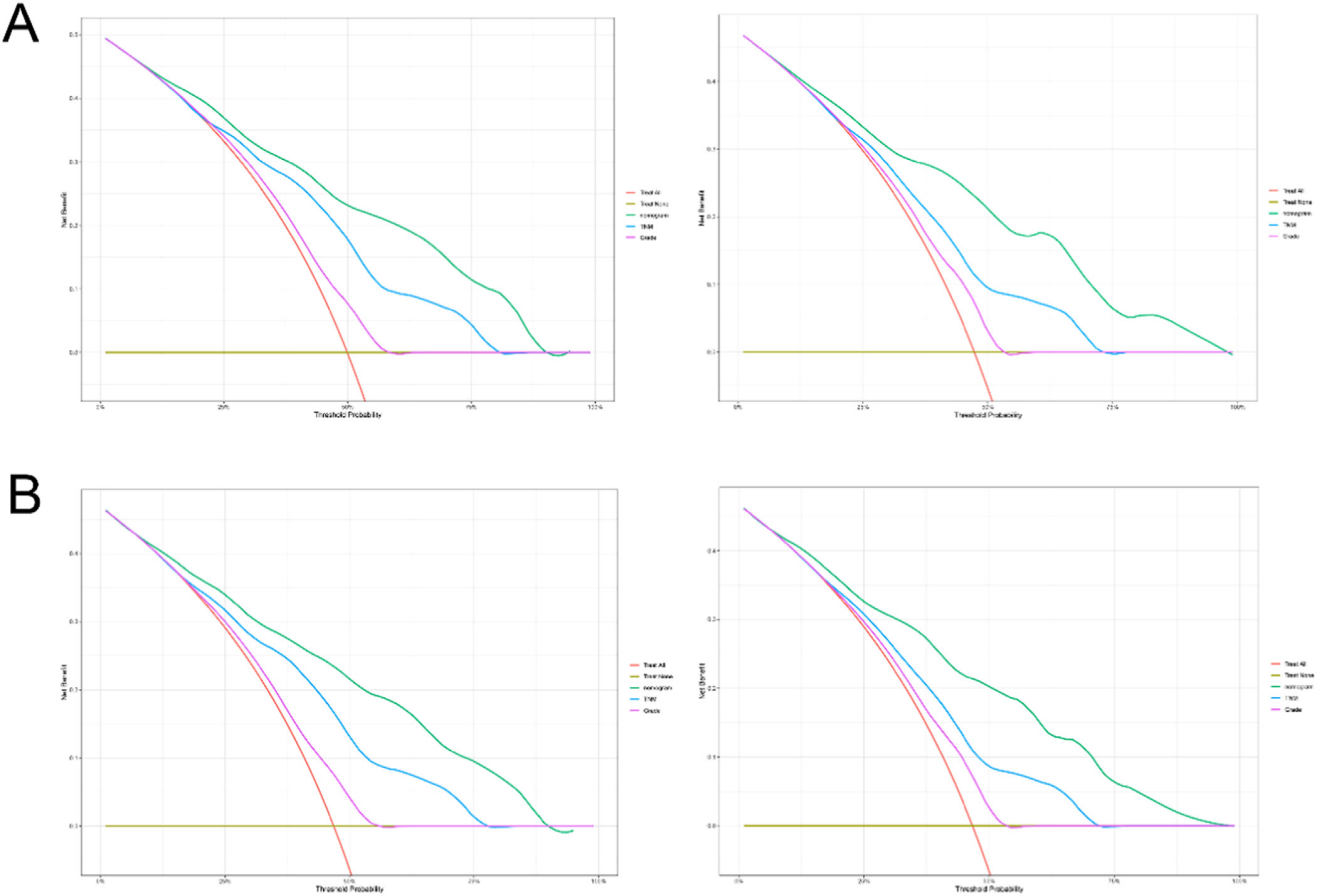
Decision curve analysis curve of the nomogram, TNM stage, and tumor grade for OS (A) and CSS (B) in the training (left) and validation cohort (right).

**Fig. 7:**
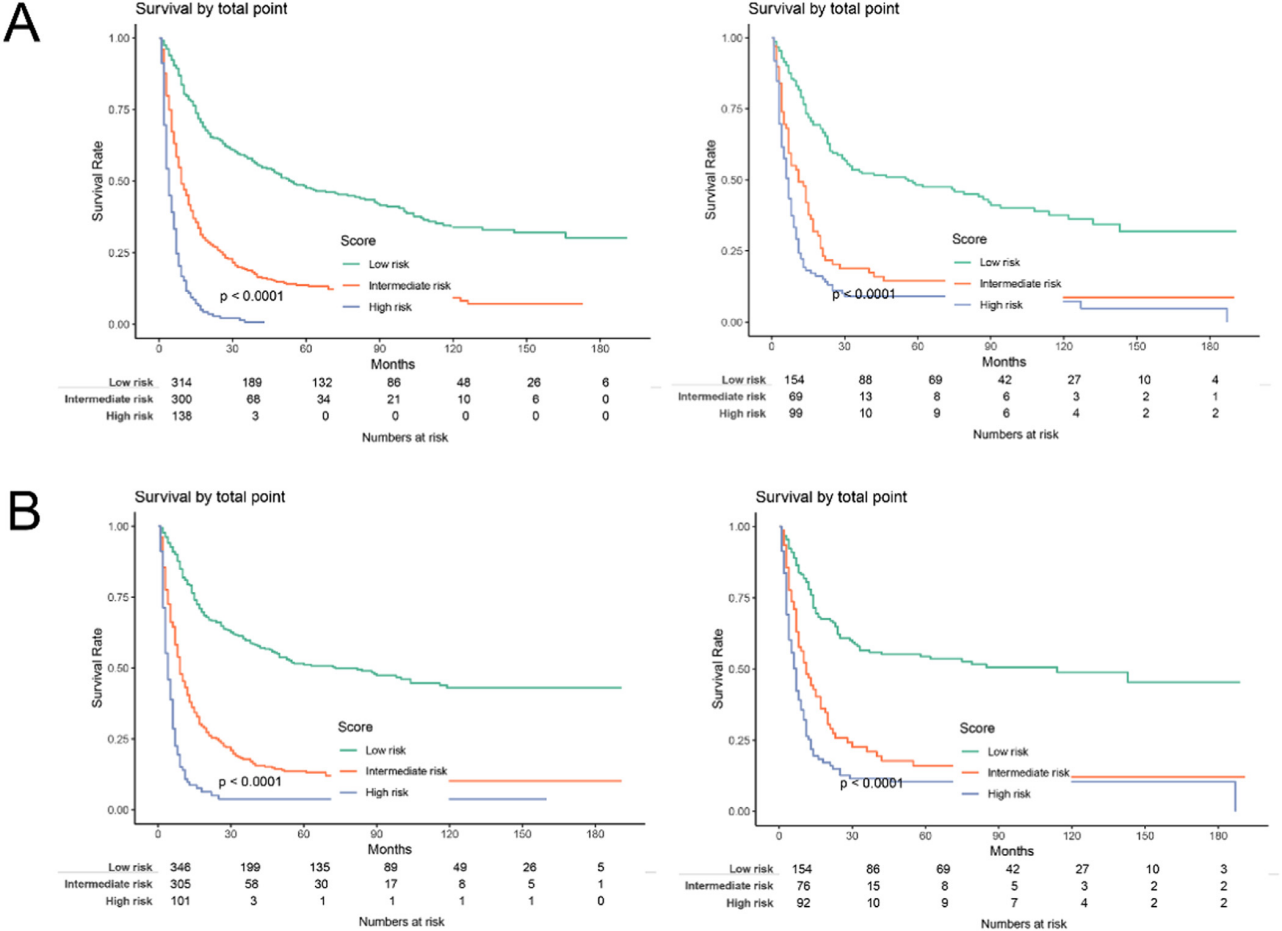
Kaplan-Meier curves of the low, intermediate, and high-risk groups in the training and validation groups for OS and CSS. (A) Kaplan-Meier curves for OS in the training (left) and validation groups (right); (B) Kaplan-Meier curves for CSS in the training (left) and validation groups (right).

The practical utility of the model was verified using the DCA.^14^ The nomogram's DCA outperformed traditional TNM staging and tumor grade in both the training and validation sets (Fig. 6). Additionally, the authors developed a new way of categorizing risks in accordance with the nomogram. Using the ideal cut-off value of the ROC, the authors determined the risk value for each patient and then separated the patients into three groups based on risk: high, intermediate, and low risks. In both the training and validation sets, patients in the high-risk group had significantly lower OS and CSS than those in the intermediate-risk and low-risk groups, according to the Kaplan-Meier curves (Fig. 7).

## Discussion

PRCC is characterized by a papillary or tubular papillary structure,^15^ and is divided into two subtypes: type 1 (characterized by monolayers of small cells with a basophilic cytoplasm and oval nuclei) and type 2 (also known as non-type 1, characterized by large pseudolayered cells and eosinophilic cytoplasm with prominent nucleoli).^16^ Previous research on pRCC has reported that it was less malignant and had higher global survival than renal clear cell carcinomas.^17^

Although the incidence of pRCC is the second highest among all types of RCC, its overall incidence is not high.^18^ Due to the low incidence of pRCC, for a single-center study, it might be challenging to gather a sufficient sample size to obtain valid results. Therefore, developing a trustworthy and precise prediction model for pRCC is crucial.^19^ The most important advantage of the SEER database is its ability to obtain clinical data from large samples, which is particularly helpful for studying rare cases.^20^ The construction of prognostic models of pRCC has been previously reported. Klatte et al. developed a prognostic nomogram to predict postoperative cancer-specific survivals in patients with pRCC.^21^ Nevertheless, the study's findings were not sufficiently valid because of the limited sample size that was used to create the nomogram. In the present study, the authors therefore developed a model capable of precisely predicting the postoperative survivals of pRCC patients, using a large sample size.

It is generally accepted that age is a critical factor in the occurrence and development of cancers. Aging increases the likelihood that genetic mutations will result in cancer.^22^ A methylation marker in kidney tissue, named TBR1, was recently identified. The results suggested that this marker gradually accumulated in renal tumors and renal metastases with age.^23^ It also highlighted the role that aging plays in the development and progression of renal cell carcinomas. Using data from a global multi-institution database, Borgmann et al. investigated the survival of 2189 patients with pRCC.^24^ The results showed that older age and poor physical status in the Eastern United States Oncology Collaboration score were significant determinants of postoperative survival of pRCC patients. Furthermore, multiple studies have reported that age played an important role in the prognoses of many tumors,^25^, ^26^, ^27^ because older patients often have multiple non-neoplastic diseases or poor nutritional status. In the present study, the authors found that OS worsened with patient age, which is consistent with the results of other studies examining how age affected clinical outcomes and prognoses of pRCC patients.

In the present research, the OS and CSS nomograms both included tumor size as an independent predictive factor. Tumor size has been shown in numerous studies to be a predictor of poor prognostic risk. According to Saha et al., there is a negative link between tumor size and survival, but a positive correlation between tumor size and pathological grade and T stage.^28^ In several studies on risk factors for distant metastasis of renal cell carcinoma, tumor size was found to be strongly associated with metastasis of renal cell carcinoma. Hutter et al. developed a nomogram for predicting lymph node metastases in patients with RCC and showed that tumor size contributed the most to predicting lymph node metastasis when compared with risk factors such as age.^29^ He later showed that tumor size also played an important role in distant metastases of renal cell carcinomas.^30^ In addition, Zastrow et al. confirmed that tumor size was a risk factor for distant metastases in patients with renal cell carcinomas, and also reported that when the tumor diameter exceeded 3 cm, the risk of distant metastasis in patients with renal cell carcinoma significantly increased.^31^]

For adjuvant therapy in pRCC patients, contrary to the findings of past investigations, the authors found that patients with metastatic or advanced pRCC had a better prognosis after radiation therapy. Ronnen et al. reported that after systemic therapy for metastatic pRCC patients, there was no significant improvement in OS at 5 years after surgery.^32^ Other studies have also revealed that pRCC was less responsive to chemotherapy and radiotherapy. The authors suspect that the reason for the difference may be that with the continuous development of medical technology, some new radiation treatment options were used. For primary and metastatic renal cell carcinomas, Stereotactic Ablative Body Radiation (SABR) is a new therapeutic option. A study by Correa et al. reported that SABR was effective in treating primary renal cell carcinomas,^33^ and Zaorsky et al. reported that stereotactic radiation treatment was both safe and effective for treating RCC oligometastases, with 90 % local control and 1 % severe toxicity.^34^ Therefore, for patients with metastatic or advanced pRCC, an appropriate radiotherapy regimen may prolong their survival, although it still needs to be proven in clinical trials.

Ultimately, the OS nomogram included the following factors: age at diagnosis, tumor size, T stage, N stage, M stage, grade, AJCC stage, and radiation. Factors included in the CSS nomogram were T stage, N stage, M stage, tumor size, AJCC stage, and radiation. In the OS and CSS nomograms, the AJCC stage and T stage, respectively, contributed most to prognosis. Additionally, the tumor's pathological grade was linked to a poorer OS. The most popular and widely accepted staging approach for different malignancies worldwide is the AJCC TNM staging system,^35^ and the Fuhrman grading system, a traditional renal cell carcinoma grading system, which is frequently used to assess the prognoses of patients with renal cell carcinomas.^36^ These findings further confirmed their important effects on predicting the survival of pRCC patients.

Although TNM staging is widely used to predict the prognoses of patients with various tumors, it ignores many factors that may affect the prognoses of patients, such as age, tumor size, and adjuvant therapy. In the present study, the authors showed that by using C-indices and AUCs, nomograms exhibited notable advantages over TNM staging and tumor grade in both validation and training sets. In addition, the nomograms were also superior to the AJCC and TNM stages in evaluating clinical applicability, as shown by the DCA. According to the nomogram, the authors categorized the patients into high, intermediate, and low-risk groups. Significant differences in OS and CSS between the three risk groups were demonstrated by the Kaplan-Meier curves. The nomogram was better than the traditional staging and grade approach at identifying the high-risk population. For patients in the high-risk group, their treatments and follow-ups in the future should receive special consideration. The nomogram also makes it easier for clinicians to implement individualized treatment and management protocols for patients. Finally, external validation was performed on adult pRCC patients at the Affiliated Hospital of Xuzhou Medical University, which demonstrated good consistency. Overall, the authors were aware of very few externally validated prior studies, which highlighted the advanced nature of the present research.

Despite the nomogram's good performance, the current study included certain drawbacks. 1) Some crucial data affecting patient survival were missing from the SEER database, including data on postoperative adjuvant treatment programs such as targeted therapy or immunotherapy, postoperative complications, laboratory test indicators, recurrence, and metastasis. 2) Postoperative pathological information on pRCC patients in the SEER database did not distinguish patients with papillary renal cell carcinoma type I from those with papillary renal cell carcinoma type II. Thus, the authors could not study them independently.

## Conclusions

The authors constructed a complete and precise nomogram to predict the 3-, 5-, and 8-year OS and CSS of adults with pRCC after identifying clinical characteristics associated with survival. The nomogram demonstrated better predictive performance when compared to the TNM stage and the tumor grade. This nomogram will help clinicians make individualized predictions of patient prognosis and create effective monitoring and follow-up strategies.

## Ethics approval and consent to participate

The studies involving human participants were reviewed and approved by Ethics Committee of the Affiliated Hospital of Xuzhou Medical University (XYFY2021-KL092–02).

## Consent for publication

Not applicable.

## Availability of data and materials

Publicly available datasets were analyzed in this study. The raw data supporting the conclusions of this article will be made available by the authors, without undue reservation.

## Authors’ contributions

Q-X.G and S.L designed the study. J-W.Z, Z-W.W and Z.L collected and analyzed the data. Q-X.G drafted the initial manuscript. H-L.L, J-Q.W and R-M.W revised the article critically. All authors approved the final manuscript.

## Funding

This study was sponsored by the 10.13039/501100010014Six Talent Peaks Project in Jiangsu Province (grant number WSW-064); a Project supported by the Natural Science Foundation of the Jiangsu Higher Education Institutions of China (grant number 19KJB310001); Post-doctoral Research Funding Project in Jiangsu Province (grant number 2021K447C); Postgraduate Research and Practice Innovation Project of Jiangsu Province (KYCX23–2926).

## Conflicts of interest

The authors declare no conflicts of interest.
